# Keap1 Negatively Regulates Transcription of Three Counter-Defense Genes and Susceptibility to Plant Toxin Gossypol in *Helicoverpa armigera*

**DOI:** 10.3390/insects15050328

**Published:** 2024-05-02

**Authors:** Xingcheng Xie, Qian Wang, Zhongyuan Deng, Shaohua Gu, Gemei Liang, Xianchun Li

**Affiliations:** 1 State Key Laboratory for Biology of Plant Diseases and Insect Pests, Institute of Plant Protection, Chinese Academy of Agricultural Sciences, Beijing 100193, China; xingchengxie@126.com (X.X.); wangqian_711@126.com (Q.W.); 2 School of Agricultural Sciences, Zhengzhou University, Zhengzhou 450001, China; dengzhongyuan@outlook.com; 3 Department of Entomology and BIO5 Institute, University of Arizona, Tucson, AZ 85721, USA; 4 Department of Entomology, China Agricultural University, Beijing 100193, China; gushaohua@cau.edu.cn

**Keywords:** *Helicoverpa armigera*, *Keap1*, counter-defense genes, gossypol, oxidative stress

## Abstract

**Simple Summary:**

The Keap1-Nrf2-ARE signaling pathway has been suggested to induce the expressions of antioxidant and counter-defense genes, and aid cells in xenobiotic and oxidative responses. Nrf2 is the central transcription factor, and Keap1 is its specific repressor in this signaling pathway. To estimate the function of the *Keap1* gene on plant toxin gossypol metabolism, we characterized the pathway in *Helicoverpa armigera*. The results demonstrated that the suppression of the *Keap1* gene not only increased the expressions of three counter-defense genes *CYP9A17*, *CYP4L11* and *UGT41B3*, but also reduced the larval mortality and promoted the larval development of those treated by the diet with gossypol. Our study showed that Keap1 negatively regulated the transcription of these three counter-defense genes and the knockdown of the *Keap1* gene contributed to decreasing the susceptibility to gossypol in the cotton bollworm, which may be beneficial for further research on the regulation of insect counter-defense gene expression and insect–plant interactions.

**Abstract:**

Expressions of a wide range of cytoprotective counter-defense genes are mainly regulated by the Keap1-Nrf2-ARE signaling pathway in response to oxidative stress from xenobiotics. Gossypol is the major antiherbivore secondary metabolite of cotton, but how the polyphagous pest *Helicoverpa armigera* copes with this phytochemical to utilize its favorite host plant cotton remains largely elusive. In this study, we first suppressed the *Keap1* gene in newly hatched larvae of cotton bollworm by feeding them the siRNA diet for 4 days. All of the larvae were subsequently fed the artificial diet supplied with gossypol or the control diet for 5 days. We identified that the knockdown of the *Keap1* gene significantly decreased larval mortality and significantly increased the percentages of larval survival, reaching the fourth instar, compared with ncsiRNA when exposed to a diet containing gossypol. Three counter-defense genes *CYP9A17*, *CYP4L11* and *UGT41B3*, which were related to the induction or metabolism of gossypol according to the report before, were all significantly up-regulated after the knockdown of the *Keap1* gene. The Antioxidant Response Elements (AREs) were also detected in the promoter regions of the three counter-defense genes above. These data indicate that the suppression of the *Keap1* gene activates the Keap1-Nrf2-ARE signaling pathway, up-regulates the expressions of counter-defense genes involved in the resistance of oxidative stress and finally contributes to reducing the susceptibility of gossypol. Our results provide more knowledge about the transcriptional regulation mechanisms of counter-defense genes that enable the cotton bollworm to adapt to the diversity of host plants including cotton.

## 1. Introduction

During over 400 million years of the coevolutionary arms race, plants have developed chemical, morphological and mechanical defenses to protect themselves from insect herbivores, while herbivorous insects have evolved various counter-defense genes to overcome plant defenses [1,2,3]. Plant secondary metabolites, also known as plant allelochemicals or toxins, are among the most diverse and effective defense weapons for plants to withstand insect attack [4]. Xenobiotic detoxification enzymes and excretion transporters such as cytochrome P450 monooxygenase (P450), carboxylesterase (CarE), glutathione S-transferase (GST), UDP-glycosyltransferase (UGT) and ATP-binding cassette (ABC) transporters are the most powerful counter-defense strategies for insect herbivores to neutralize antiherbivore plant allelochemicals [5,6].

Host generalists have easy access to abundant sources of edible plants while facing the serious challenge of the diverse and unpredictable plant defenses of potential host plants [5,7]. One such host generalist is the cotton bollworm (CBW), *Helicoverpa armigera*, a notorious crop pest capable of feeding on over 180 plant species belonging to at least 68 families [8,9]. Cotton is among the common and favorite host plants of this insect species, although it produces gossypol as part of its defense. Generalists may have fewer limitations of food availability, but they have to deal with the diversity and unpredictability of plant defenses and potential pathogens [5,7]. The expansion of gene families associated with detoxification and the transport of plant defense allelochemicals [5,10,11] and the functional versatility of such counter-defense genes [7] assist generalists in coping with the challenge of diverse and unpredictable plant defenses. In addition, the evolution of a more efficient immune system is also proposed to protect generalists against a broader range of potential pathogens [12]. 

Gossypol is the main polyphenolic compound of cotton plants, produced by pigment glands in cotton seeds, leaves, stems, roots and flower buds [13]. Gossypol contributes to some toxic effects in vertebrates, but it enhances the insecticidal activities of cotton plants [14]. Gossypol was reported to inhibit some enzyme activity such as lipid peroxidase and protease in *Spodoptera littoralis* larvae [15]. Different concentrations of gossypol in artificial diets also exhibited different effects on the development of the cotton bollworm [16]. Cotton pigment glands and higher levels of gossypol resulted in a significant decrease in larval weight and the moth eclosion rate of the cotton bollworm, and also delayed the development of larvae and pupae [17]. The reactive toxicity of gossypol is closely related to the phenolic hydroxyl and aldehyde groups in the molecule [18]. Therefore, gossypol is also considered one kind of natural insecticides. Gossypol is possibly involved in oxidative stress and exhibits pro-oxidative effects [19]. Recent studies have demonstrated that gossypol exposure increased the level of reactive oxygen species (ROS), induced oxidative stress and resulted in mitochondrial dysfunction during mouse oocyte [13]. It has also been shown that gossypol-induced ROS production had antitumor effects via mitochondrial apoptosis in human colorectal carcinoma cells [20]. In addition, gossypol affected the mitochondrial respiratory chain, inhibited electron transport and stimulated ROS generation in *Yarrowia lipolytica* [21].

The cotton bollworm is able to tolerate gossypol due to its complicated and flexible metabolic detoxification system [22]. But the strategy of how to cope with gossypol when feeding on cotton plants is still unknown. It has been previously observed by using microarray analysis that the *CYP9A17* gene in the cotton bollworm was up-regulated in response to both the treatment of cotton leaf and square, while the *CYP4L11* gene was up-regulated in response to both the treatment of cotton leaf and boll compared with the control diet [23]. It is important to note that the relative expression of the *CYP4L11* gene was overexpressed in *H. armigera* strains with different levels of resistance to the insecticide deltamethrin [24]. The *CYP9A17* gene was induced by 0.1% gossypol and related to deltamethrin tolerance in the cotton bollworm [25]. Feeding the cotton bollworm with the glanded cotton leaves also slightly induced *CYP9A17* gene expression compared with the glandless leaves [25]. The transcriptome response of *H. armigera* to different host plants showed that feeding treatments containing cotton leaves up-regulated the relative expression of the *CYP4L11* gene in comparison with corn fruits, soybean leaves, chili fruit and the artificial diet [26]. The *CYP4L11* gene was about 20-fold and 8-fold up-regulated with the feeding treatments of 0.16% gossypol and cotton leaves compared to the artificial diet, respectively [26]. The *UGT41B3* gene was also estimated to partially metabolize gossypol through glycosylation in the cotton bollworm [27]. Taken together, three counter-defense genes *CYP9A17*, *CYP4L11* and *UGT41B3* can be induced or can directly metabolize gossypol, but the corresponding molecular mechanism by which the cotton bollworm utilizes cotton as a host plant is still unknown.

Xenobiotic and oxidative responses defend cells against external and internal toxicities [28]. The Keap1-Nrf2-ARE signaling pathway is the principal inducible defense against oxidative and electrophilic stresses by regulating cytoprotective gene expressions [29,30]. Nuclear factor E2-related factor 2 (Nrf2) is a transcription factor of the basic leucine zipper (bZIP) family and can regulate the basal and stress-inducible activation of cytoprotective gene expressions [31,32]. Cap ‘n’ collar isoform C (CncC), the homolog of the mammalian Nrf2 in invertebrates, has been identified as the central regulator of antioxidant and detoxification genes [33]. CncC can protect the organism from oxidative stress by regulating many stress-responsive genes and be involved in the resistance against insecticides or xenobiotics [34]. 

Kelch-like ECH-associated Protein 1 (Keap1) is the specific repressor of CncC. Under physiological conditions, CncC is immobilized by the inhibitor Keap1 in the cytoplasm [35]. Keap1 contributes to directly triggering the ubiquitin-dependent proteasomal degradation of CncC [36]. However, Keap1 can also function as a sensor of oxidants and electrophiles attributed to the structure of redox-sensitive cysteine residues [37,38]. Upon exposure to oxidative stresses or electrophilic xenobiotics, Keap1 is firstly oxidized and the inhibition of CncC by Keap1 is abolished [39], leading to the subsequent stabilization and nuclear translocation of CncC [40]. Then, CncC forms a heterodimer through the integration with the muscle aponeurosis fibromatosis (Maf) protein [41], finally binds to the Antioxidant Response Elements (AREs) in the promoter regions of the target antioxidant genes [42] and induces the expression of these genes [43].

As a new biotechnology tool, RNA interference (RNAi) has been widely used for research on gene functions. Identifying gene functions by silencing gene expression is a promising strategy for pest control [44]. The Keap1-Nrf2-ARE signaling pathway is regarded as playing a crucial role in a wide range of detoxification gene expressions in phase I, phase II and phase III [34,45]. Hence, in view of the effects of gossypol exposure on other organisms [13,20,21], one hypothesis is that gossypol initiates oxidative stress, stimulates the generation of ROS and activates the Keap1-Nrf2-ARE signaling pathway in *H. armigera*. In order to determine whether the above gossypol-related counter-defense genes *CYP9A17*, *CYP4L11* and *UGT41B3* in *H. armigera* can be regulated by the Keap1-Nrf2-ARE signaling pathway, the *Keap1* gene was suppressed using the method of RNAi by feeding the diets mixed with siRNA in this study. The effects of RNAi have also been evaluated through the relative expression of the *Keap1* gene after the suppression and the diet bioassays treated with 5% (*w*/*w*) gossypol. After the guarantee of successful suppression, the relative expressions of the *CYP9A17*, *CYP4L11* and *UGT41B3* genes were detected. Finally, the conserved elements, AREs, of these three genes in the 5′ flanking region were also analyzed.

## 2. Materials and Methods

### 2.1. Insect Rearing 

The laboratory colony of *H. armigera* used in this study was established with more than 1000 larvae originally collected from Xuchang City (Henan, China) in June 2016. The colony was then maintained in the growth cabinet at 27 ± 1 °C with a photoperiod of 16 h of light and 8 h of darkness. The relative humidity of the growth cabinet for the larvae and the adults was kept at 40 ± 10% and 70 ± 10%, respectively. The larvae were fed the artificial diets containing soybean and corn flour, whereas the adults were provided with 10% honey water for mating and oviposition.

### 2.2. Suppression of Keap1 Gene and Diet Bioassays 

Two siRNAs (siRNA1 and siRNA2) targeting the gene *Keap1* (GenBank: KU355788.1; XM_021340555.1; XM_021340556.1) and one non-targeting negative control siRNA (Table 1) were designed and synthesized by RiboBio (Guangzhou, China). All of these siRNAs in the same chemical modification of 2′-O-methylation for enhanced efficacy and stability [46] were firstly dissolved in DEPC-treated water at a concentration of 20 μM. We balanced the mixed siRNA1 and siRNA2 solution and recorded the mixed liquor as siRNA-Keap1. We subsequently mixed 40 mL of the diet with 400 μL of the siRNA-Keap1 or siRNA-NC solution. The final concentrations were 100 nmol/L of siRNA1 and siRNA2 equally or 200 nmol/L of siRNA-NC. We individually dispensed each siRNA diet into 40 wells (1 mL diet per well) of a 24-well bioassay tray. Diets treated with 5% (*w*/*w*) gossypol (Ekear, Shanghai, China) or lacking gossypol were made by gently adding gossypol or an equal quantity of water to the soybean and corn flour diet. We separately transferred 1 mL 5% (*w*/*w*) gossypol or the control diet into each well of the bioassay tray.

A total of 400 newly hatched larvae were carefully divided into groups fed with 2 kinds of siRNA diets (5 larvae per well). After 4 days of siRNA diet treatments, 45 larvae (3 biological replicates of 15 larvae each) were randomly obtained from each treatment. All of these 45 larvae were immediately flash-frozen in liquid nitrogen and stored at −80 °C until RNA extraction. A total of 60 larvae (3 biological replicates of 20 larvae each) feeding on each siRNA diet were placed into wells containing 5% (*w*/*w*) gossypol or the control diet (1 larvae per well). In total, there were four treatments (two siRNAs × two diets). After 5-day feeding experiments, the number of survivors and their developmental stage were recorded.

### 2.3. RNA Extraction and cDNA Synthesis

Three RNA samples for each siRNA treatment were extracted by using TRIzol reagent (Invitrogen, Carlsbad, CA, USA) according to the manufacturer’s manual. The total RNA was treated with DNase I (New England Biolabs, Beverly, MA, USA) for 10 min to remove the potential gDNA contamination, then cleaned with phenol/chloroform extraction and finally dissolved in DEPC-treated water. The concentrations of the RNA samples were measured by using a NanoDrop 2000 spectrophotometer (Thermo Scientific, Waltham, MA, USA). A total of 1 μg of cleaned RNA for each siRNA treatment was reverse-transcribed with oligo(dT)_20_, RNase Inhibitor and M-MuLV Reverse Transcriptase (New England Biolabs, Beverly, MA, USA).

### 2.4. RT-qPCR Analysis of Keap1 Gene Expression

Quantitative RT-PCR (RT-qPCR) of the *Keap1* gene and two internal reference genes, *β-Tubulin* (GenBank: JF767013.1) and ribosomal protein L-32 *RPL32* (GenBank: JQ744274.1), was individually performed in 20 µL reactions containing 3 µL cDNA, 10 µL 2× GoTaq^®^ qPCR Master Mix (Promega, Madison, WI, USA), 1 μL each of forward and reverse gene-specific primers (10 μM), 0.2 µL supplemental CXR reference dye and 4.8 µL nuclease-free water. The primer pairs of the two reference genes (*β-Tubulin* and *RPL32*) were the same as those of Zhang et al. [47] and the other primer pairs for RT-qPCR analysis were designed by Beacon Designer 7 (Table 1).

The RT-qPCR for each sample was conducted with three technical replicates and three biological replicates by using the CFX Connect Real-Time PCR Detection System (Bio-Rad, Hercules, CA, USA). The cycling program of RT-qPCR was under the following conditions: 95 °C for 2 min, followed by 40 cycles of 95 °C for 15 s and 60 °C for 1 min, during which period real-time data were obtained. Melting curve analysis was carried out from 65 °C to 95 °C for the *Keap1* gene and two reference genes to ascertain the specific amplifications. The amplification efficiency (E) of each gene was obtained by using the formula E = 10^−1/slope^ − 1 with the log template concentration (*x*-axis)–Ct value (*y*-axis) line. The expression level of each gene was calculated with their mean Ct and amplification efficiency, and further normalized with the geometric mean of the expression of the two reference genes.

Expression level = (1 + E_gene_)^−Ct^

Normalized expression level of target gene = $\frac{{\left(1+E_{\mathrm{target}\;\mathrm{gene}}\right)}^{-{\mathrm{Ct}}_{\mathrm{target}\;\mathrm{gene}}}}{\sqrt{{\left(1+E_{\beta \text{-}\mathrm{Tub}}\right)}^{-{\mathrm{Ct}}_{\beta \text{-}\mathrm{Tub}}}\times {\left(1+E_{\mathrm{RPL}32}\right)}^{-{\mathrm{Ct}}_{\mathrm{RPL}32}}}}$

### 2.5. RT-qPCR Analysis of Down-Stream Target Genes Expression

To identify whether *CYP9A17* (GenBank: AY753201.1), *CYP4L11* (GenBank: KM016726.1) and *UGT41B3* (GenBank: JQ070217.1) were regulated by the *Keap1* gene, the transcriptional levels of these three candidate target genes were assessed after the suppression of the *Keap1* gene. cDNA samples and the RT-qPCR program were performed as described earlier. The only difference for the *CYP4L11* gene was annealing and extension at 55 °C for 1 min, but not at 60 °C.

### 2.6. Promoter Analysis of Three Target Genes

In order to explain the regulation mechanism of the three counter-defense genes after the knockdown of the *Keap1* gene in the cotton bollworm, the promoter regions of these target genes were analyzed. The genomic sequences of these target genes were obtained from the genome of *H. armigera* (GenBank: GCA_002156985.1). The sequences of 2000 nucleotides located up-stream of the transcription start site (TSS) were used to search for the conserved elements, AREs. The following consensus sequence was used to identify the conserved elements of expression regulation in the promoter regions: ARE motif sequence (5′-TMANNRTGAYNNNGCR-3′) [48].

### 2.7. Statistical Analysis

The independent *t*-test was used to estimate the effects of siRNA-Keap1 and siRNA-NC on the expression levels of the *Keap1* gene after suppression. Two-way ANOVA was performed to examine the impacts of siRNAs, diets and their interactions on the percentages of larvae reaching the fourth instar and the percentages of mortality after the arcsine square root transformation. The independent *t*-tests were conducted to test the significance of differences in the percentages of larvae reaching the fourth instar and the percentages of mortality between the two siRNAs treatments within the same diet or the two diet treatments within the same siRNA. The independent *t*-tests were also conducted to determine the effects of silencing the *Keap1* gene on the expressions of the three counter-defense genes *CYP9A17*, *CYP4L11* and *UGT41B3*.

## 3. Results

### 3.1. Knockdown of the Keap1 Gene Significantly Reduced Its Expression

**Figure 1:**
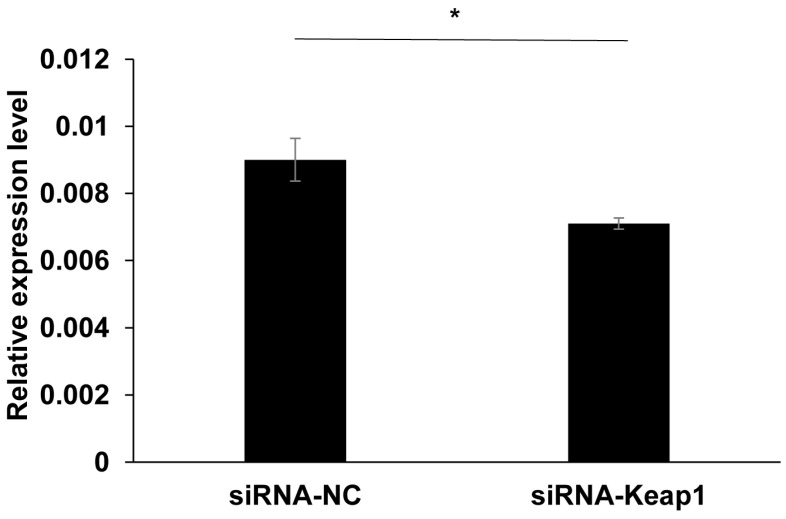
The effects of siRNA on the transcription level of the *Keap1* gene. The data and error bars represent the means ± standard error of *Keap1* gene expression based on three biological replicates of three technical repeats for each treatment with siRNA. One asterisk indicates the significant differences at the *p* < 0.05 level based on the independent *t*-test.

To reveal the function of the *Keap1* gene, RNAi was used to knockdown the *Keap1* gene in *H. armigera*. The newly hatched larvae were fed for 4 days on diets supplemented with small interfering RNA of the *Keap1* gene (100 nmol/L of siRNA1 and siRNA2 equally) or 200 nmol/L of the negative control siRNA (siRNA-NC). Actually, siRNA-Keap1 was a 1:1 mixture of siRNA1 and siRNA2 targeting the *Keap1* gene at the same concentration of 100 nmol/L. The real-time PCR results showed that *Keap1* expression could be significantly reduced by 22% in comparison with siRNA-Keap1 and siRNA-NC (*p* = 0.045, independent *t*-test) (Figure 1).

### 3.2. Suppressing the Keap1 Gene Decreased the Susceptibility of H. armigera to Gossypol 

After siRNA treatments, the larvae were immediately fed the diet containing 5% (*w*/*w*) gossypol or the control diet for the 5-day bioassay experiment. The results indicated that the down-regulation of the *Keap1* gene could reduce the gossypol-induced larval mortality and accelerate their growth.

**Figure 2:**
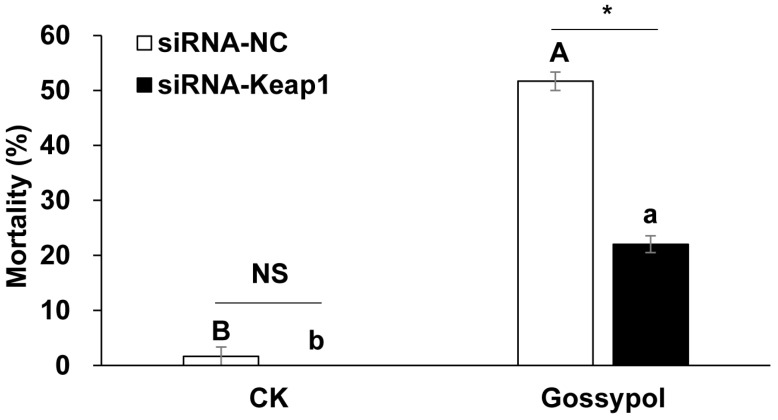
The effects of the *Keap1* gene silencing on the gossypol-induced larval mortality of *Helicoverpa armigera*. The data and error bars represent the means ± standard error of the larval mortality for each treatment. After the arcsine square root transformation, the larval mortality are used for significance analysis. Different letters indicate significant differences at the *p* < 0.05 level based on the independent *t*-test for the same siRNA treatment (lower case letters indicate siRNA-Keap1 treatment and capital letters represent siRNA-NC treatment). One asterisk indicates significant differences at the *p* < 0.05 level based on the independent *t*-test for the same diet treatment. “NS” means there is no significant difference for the same diet treatment.

Two-way ANOVA showed significances on siRNAs (*F* = 24.208, *p* = 0.001), diets (*F* = 235.809, *p* = 0.000) and their interactions (*F* = 9.121, *p* = 0.017) for larval mortality. For the pre-fed siRNA-Keap1 treatment group, there was a significant difference between feeding the diet containing gossypol (22.02%) and the CK diet (0%) in larval mortality (*p* = 0.001, independent *t*-test). Regarding the pre-fed siRNA-NC treatment, there was also a significant difference between feeding the diet containing gossypol (51.67%) and the CK diet (1.67%) in larval mortality (*p* = 0.008, independent *t*-test) (Figure 2). On the diets containing gossypol, compared with the treatment group pre-fed siRNA-NC, suppressing the *Keap1* gene by using the pre-fed siRNA-Keap1 treatment could significantly decrease larval mortality (*p* = 0.000, independent *t*-test). On the contrary, there was no significant difference between the mortality of larvae pre-fed with the siRNA-Keap1 and siRNA-NC treatments on the CK diets (*p* > 0.05, independent *t*-test) (Figure 2). 

**Figure 3:**
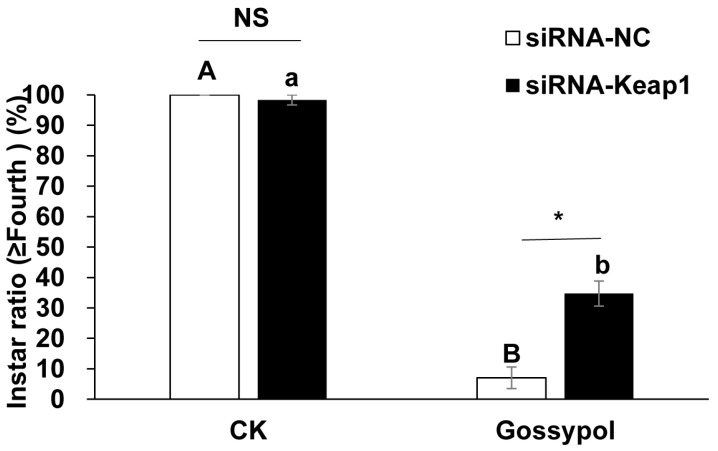
The effects of the *Keap1* gene silencing on the gossypol-induced larval growth retardation of *Helicoverpa armigera* survivors. The data and error bars represent the means ± standard error of the percentages of larvae reaching the fourth instar for each treatment. After the arcsine square root transformation, the percentages of larvae reaching the fourth instar are used for significance analysis. Different letters indicate significant differences at the *p* < 0.05 level based on the independent *t*-test for the same siRNA treatment (lower case letters indicate siRNA-Keap1 treatment and capital letters represent siRNA-NC treatment). One asterisk indicates the significant differences at the *p* < 0.05 level based on the independent *t*-test for the same diet treatment. “NS” means there is no significant difference for the same diet treatment.

To determine whether the down-regulation of the *Keap1* gene affects the larval growth and development of survivors, the percentages of larvae reaching the fourth instar were calculated. Two-way ANOVA showed siRNAs (*F* = 5.621, *p* = 0.045), diets (*F* = 248.995, *p* = 0.000) and their interactions (*F* = 11.839, *p* = 0.009) all had significant effects on larval growth. For the pre-fed siRNA-Keap1 treatment, there was a significant difference between those fed the diet containing gossypol (34.72%) and those fed the CK diet (98.33%) in the percentages of larvae reaching the fourth instar (*p* = 0.001, independent *t*-test). For the pre-fed siRNA-NC treatment, there was also a significant difference between those fed the diet containing gossypol (7.04%) and those fed the CK diet (100%) in the percentages of larvae reaching the fourth instar (*p* = 0.007, independent *t*-test) (Figure 3). On the diets containing gossypol, the treatment group pre-fed the siRNA-Keap1 treatment (34.72%) had a significantly greater percentage of larvae reaching the fourth instar than did the treatment group pre-fed siRNA-NC (7.04%) (*p* = 0.026, independent *t*-test). By contrast, no significant difference in the percentages of larvae reaching the fourth instar was found between the larvae pre-fed the siRNA-Keap1 (98.33%) and those pre-fed siRNA-NC (100%) on the CK diets (*p* > 0.05, independent *t*-test) (Figure 3). 

### 3.3. Silencing the Keap1 Gene Resulted in Up-Regulation of Three Target Genes 

**Figure 4:**
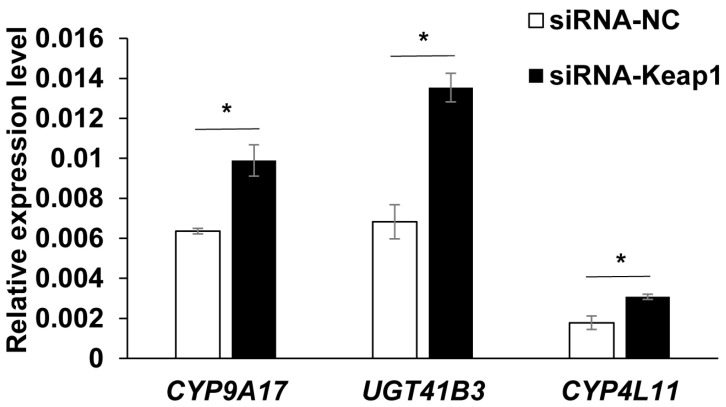
The effects of the *Keap1* gene silencing on the transcription level of down-stream detoxification genes. The data and error bars represent the means ± standard error based on three biological replicates of three technical repeats for each treatment with siRNA. One asterisk indicates the significant differences at the *p* < 0.05 level based on the independent *t*-test.

The Keap1-Nrf2-ARE pathway can regulate a large number of detoxification enzyme genes such as P450s, GSTs, CarEs, UGTs and ABCs. Among the detoxification enzyme genes, three target genes *CYP9A17*, *CYP4L11* and *UGT41B3* which had been reported to be related to the metabolism or transport of the plant allelochemical gossypol were selected to ascertain the effects of silencing the *Keap1* gene on their expressions. Compared to siRNA-NC treatment, these three genes *CYP9A17*, *CYP4L11* and *UGT41B3* were all significantly up-regulated after the knockdown of the *Keap1* gene. The up-regulation fold changes for the target genes *CYP9A17*, *CYP4L11* and *UGT41B3* were 1.56 (*p* = 0.011, independent *t*-test), 1.73 (*p* = 0.023, independent *t*-test) and 1.98 (*p* = 0.004, independent *t*-test), respectively (Figure 4). 

### 3.4. Identification of AREs in the Promoter Regions of the Target Genes

**Figure 5:**
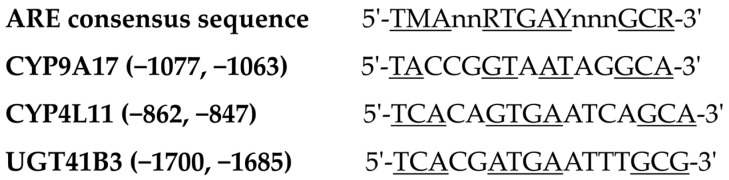
Sequence analysis of predicted Antioxidant Response Elements (AREs) in the promoter regions of three detoxification genes. The consensus ARE sequence of mammalian detoxification genes is identified as 5′-TMAnnRTGAYnnnGCR-3′, in which M, R, Y and n represent A/C, A/G, C/T and A/T/G/C, respectively. The conserved nucleotides in the ARE-like elements are underlined. The ARE motif position is defined in relation to the transcription start site (TSS, +1), and the up-stream sequence of TSS is marked “−”.

It is estimated that *CYP9A17*, *CYP4L11* and *UGT41B3* are regulated by the Keap1-Nrf2-ARE signaling pathway. Once we confirmed that these three genes were down-regulated after the suppression of the *Keap1* gene, the corresponding motifs in the promoter regions of these genes were then analyzed. The results showed that ARE motifs were located in the promoter regions of these target genes (Figure 5).

## 4. Discussion

It has been previously observed that arrays of antioxidant and detoxification genes can be regulated by the activation of the Keap1-Nrf2-ARE signaling pathway [43,49,50]. To determine the functions of the *Keap1* gene in the cotton bollworm, we knocked it down by feeding the diets mixed with siRNAs in this study. The treatment with siRNA-Keap1 significantly reduced the expression of the *Keap1* gene compared to the treatment with siRNA-NC, and the reduction was more than 20%.

The polyphagous pest, the cotton bollworm, feeds on a variety of host plants including its favorite host plant, cotton. In this study, we chose gossypol as the extraneous oxidative stress of larval cotton bollworm, because gossypol is the main plant allelochemical of cotton. The larvae pre-fed siRNAs showed significant impacts in terms of both larval mortality and development upon exposure to the diet supplied with 5% (*w*/*w*) gossypol or the control diet. For the diets containing gossypol, the treatment group pre-fed siRNA-Keap1 significantly decreased larval mortality compared to the treatment group pre-fed siRNA-NC and significantly increased the percentages of surviving larvae reaching the fourth instar compared to siRNA-NC treatmentat the same time. Therefore, it is obvious that the suppression of the *Keap1* gene can reduce the susceptibility of the cotton bollworm to gossypol and increase its tolerance to oxidative stress. However, which counter-defense gene may be involved in the process of the response to gossypol regulated by the Keap1-Nrf2-ARE signaling pathway was still not definite.

Previous studies have suggested that three counter-defense genes *CYP9A17*, *CYP4L11* and *UGT41B3* in the cotton bollworm were induced by gossypol or related to the metabolism of it [23,25,26,27]. The relative expression of the *CYP4L11* gene in the cotton bollworm was up-regulated with the feeding treatments of 0.16% gossypol and cotton leaves compared to the artificial diet, but the growth rate of third instar cotton bollworm feeding on gossypol diets was reduced after the down-regulation of the *CYP4L11* gene by using RNAi [26]. These data suggest that the *CYP4L11* gene is engaged in gossypol detoxification in the cotton bollworm. The enzymatic assays with the heterologously expressed UGT41B3 showed that gossypol was partially metabolized to the diglycosylated gossypol isomer 5 by UGT41B3 [27]. This demonstrates that *UGT41B3* is another important counter-defense gene for gossypol detoxification in the cotton bollworm.

To estimate whether these three detoxification genes were regulated by the signaling pathway of Keap1-Nrf2-ARE, the transcriptional levels of the above target genes were also obtained after the knockdown of the *Keap1* gene. The siRNA-Keap1 treatment had significant effects on the gene expression of *CYP9A17*, *CYP4L11* and *UGT41B3*, and the up-regulation fold changes were 1.56, 1.73 and 1.98, respectively. In addition, the up-regulation fold changes of these three genes were all greater than the reduction in the *Keap1* gene due to siRNA-Keap1 and siRNA-NC treatments. This confirms that the suppression of the *Keap1* gene up-regulates the transcription of these three detoxification genes, but is not directly affected by siRNA-Keap1 treatment. In other words, the counter-defense genes *CYP9A17*, *CYP4L11* and *UGT41B3* are the down-stream genes of the Keap1-Nrf2-ARE signaling pathway and are negatively regulated by the *Keap1* gene.

ROS are generated in a large number of physiological and metabolic processes [51]. Excessive ROS accumulations contribute to the oxidative damage of nucleic acids, lipids and proteins [52]. However, ROS have also been found to serve as the signaling molecules to the response of oxidative stress [42,53]. Polyphagous herbivorous insects can feed on a vast diversity of hosts in complex plant communities. In order to cope with the complexity of the environment, the insects have to be frequently exposed to many environmental stressors such as plant allelochemicals, herbicides and insecticides [54]. So, the insects are unavoidably subjected to the different oxidative stresses elicited by ROS generation [55]. 

The Keap1-Nrf2-ARE pathway is one of the key signaling pathways involved in activating the stress response and inducing the expressions of antioxidant and detoxification genes for resistance [56]. Superoxide dismutase (SOD) enzymes play a crucial part in removing ROS and antioxidant defense [57]. Overexpression of the *CncC* gene up-regulated the transcriptional level of the *SOD* gene in *Spodoptera frugiperda* cells, but knockdown of the *CncC* gene significantly decreased the transcription and activity of the *SOD* gene both in Sf9 cells and larvae [58]. Flavone exposure significantly boosted H_2_O_2_ content in the larval midgut of the cotton bollworm, but knockdown of the *CncC* gene significantly decreased the flavone-induced *CYP321A1* gene expression and resulted in the lower flavone tolerance in *H. armigera* [59]. The expression level of the *CYP6DA2* gene was significantly induced by gossypol, but RNAi of the *CncC* gene was able to significantly reduce the transcript of the *CYP6DA2* gene and decrease the tolerance to gossypol in cotton aphids [60]. The transcription factor *Nrf2* increased *GSTe1* gene expression in response to ROS induced by the phytochemicals and insecticides, but the suppression of the *Nrf2* gene significantly decreased the xenobiotic-induced expressions and antioxidative activities of *GSTs* in *Spodoptera litura* [61]. 

The expression of the *GSTd1* gene in *Drosophila melanogaster* was increased by *Keap1* knockdown after 4 days of treatment but tended to recover after 10 days [31]. Keap1 loss-of-function mutations also extended the lifespan of *Drosophila* males and increased the paraquat resistance [31]. Another study showed that RNAi of the *Keap1* gene activated the Keap1-Nrf2-ARE pathway in *Drosophila* and sufficiently conferred resistance to the lethal effects of the pesticide malathion [43]. These results above indicate that the deficiency of Keap1, leading to the accumulation of CncC, subsequently facilitated the expression of numerous detoxification genes. The RNA-Seq results also demonstrate that overexpression of the *Keap1* gene in *Drosophila* Kc cells significantly decreased the expression of many P450 genes under the stress exposure of deltamethrin [62]. In addition, *Nrf2* gene overexpression in *Drosophila* Kc cells showed higher cell survival and expression of detoxification enzymes than *Keap1* gene overexpression and *Nrf2* gene knockdown under deltamethrin stress [63]. It is certain that the Keap1-Nrf2-ARE pathway can regulate the transcriptions of various antioxidant and detoxification genes of phase I, II and III in a wide range of insects [49]. A recent study also suggests that the knockout of the *Keap1* gene by the CRISPR/Cas9 strategy drastically decreased ROS content in Sf9 cells, significantly increased the enzyme activity of P450s, CarEs and GSTs, and strengthened the tolerance of Sf9 cells to xenobiotics indole 3-carbinol (I3C) and methoprene [64]. But other transcription factors and signaling pathways are also likely to be involved in the regulation of xenobiotic-induced gene expressions in response to oxidative stress [65,66]. Therefore, further studies are necessary to elucidate the molecular mechanisms of antioxidant and detoxification gene expressions with regard to the xenobiotic metabolism.

The ARE motifs are important *cis*-acting elements for the regulation of detoxification enzyme genes against oxidative stress [67]. The ARE sequence of the *CYP6B1* gene identified in *Papilio polyxenes* was (−137/−128) ATGACTGGCA (2N between the conserved “RTGAY” and “GCR” motifs), and mutation of this element abolished both the basal and xanthotoxin-induced expression of the *CYP6B1* promoter in Sf9 cells [68]. There were two AREs (−1940/−1930) ATGACTTTGCA (3N between the conserved “RTGAY” and “GCR” motifs) and (−191/−181) ATGACTCAGCA for CncC/Maf binding to the *CYP321A8* gene in *S. exigua*, and mutations of the two CncC/Maf binding sites decreased the expression of the *CYP321A8* gene induced by these transcription factors [69]. Along the same line, the expressions of the *CncC* and *Maf* genes in Sf9 cells promoted the transcription of PGL3-GSTe6 derived from *S. exigua*, while the mutation of the CncC/Maf binding sequence (−190/−176) AATGACAAGGCAAA in the *GSTe6* promoter region reduced the transcription activity [70]. In addition, mutational analysis of the ARE (−87/−78) ATGATTCGCA also indicated that this ARE was essential for the basal and flavone-induced expression of the *CYP321A1* gene in *H. zea* [71]. In this study, the ARE-like elements were identified in the promoter regions of the counter-defense genes *CYP9A17*, *CYP4L11* and *UGT41B3* in the cotton bollworm. All of these three genes consisted of the ARE-like element [48,72]. This evidence also strongly indicated that these target genes were regulated by the Keap1-Nrf2-ARE pathway. Interestingly, the ARE-like elements found in the detoxification genes *CYP4L11* and *UGT41B3* were better matched with the ARE consensus sequence compared to the *CYP9A17* gene, which explained why the up-regulation fold changes of the *CYP4L11* and *UGT41B3* genes were higher than those of the *CYP9A17* gene.

In this study, we demonstrated that the suppression of the *Keap1* gene increased the expression levels of three counter-defense genes and contributed to reducing the susceptibility of gossypol in the cotton bollworm. The results from this study indicate that as the specific repressor of CncC, it is likely that Keap1 is the most important regulator of detoxification gene expression in the cotton bollworm to enable it adapt to the diversity of host plants including cotton. Our results provide further understanding of the functions of the Keap1-Nrf2-ARE signaling pathway in gossypol metabolism in the cotton bollworm, laying a theoretical foundation for developing new environmentally friendly strategies for integrated pest management.

## Figures and Tables

**Table 1:** Primer pairs and siRNAs used for expression analysis of target or reference genes.

Primer	Sequence (5′-3′)	Amplification Efficiency
Keap1-F	TTCATCTTACGACAGCGATT	97.45%
Keap1-R	TCCATTACAGCAACTCCTAC	
β-TUB-F	AGCAGTTCACCGCTATGTTC	96.69%
β-TUB-R	AGGTCGTTCATGTTGCTCTC	
RPL32-F	CATCAATCGGATCGCTATG	99.47%
RPL32-R	CCATTGGGTAGCATGTGAC	
CYP9A17-F	TCCGCCAGGTCTATTCCC	96.06%
CYP9A17-R	ACCAACTCCTTGATGAAT	
CYP4L11-F	CGCTAATATAACTGCTCTT	105.31%
CYP4L11-R	ACCTTCATCGTCTATCTT	
UGT41B3-F	TACCACAAGTATAGCAGTAGC	94.17%
UGT41B3-R	CAAGATGGCGTGATAGTTC	
siRNAs		
Keap1 siRNA1 sense	GCTGTAATGGACGGACTAT	N.A.
Keap1 siRNA2 sense	GCACGTCGTTCCTAGACAT	N.A.
ncsiRNA sense	siM12921102701 (kept confidential according to RiboBio terms)	N.A.

N.A.: not applicable.

## Data Availability

The data presented in this study are available on request from the corresponding author.
